# Effects of different levels of physical activity on arterial stiffness and physical fitness performance in firefighters

**DOI:** 10.7150/ijms.96269

**Published:** 2024-08-01

**Authors:** Chun-Hao Chang, Yi-Ju Hsu, Chih-Hsien Huang

**Affiliations:** 1 Graduate Institute of Sports Science, National Taiwan Sport University, Taoyuan City, Taiwan.; 2 Shulin Branch Fifth Corps, Fire Department, New Taipei City Government, New Taipei City, Taiwan.

**Keywords:** firefighter, arteriosclerosis, body composition, physical training

## Abstract

**Background:** Firefighters have lower levels of physical activity while on call. It is critical to understand the impact of firefighters' physical activity on arterial stiffness. This study classified groups by physical activity level and combined peripheral vascular monitor measurement to explore the relationships between the level of physical activity and cardiovascular (CV) risk and physical fitness (PF) of firefighters, as well as the acute response to arterial stiffness (AS) following maximal aerobic exercise test (MAET) intervention.

**Methods:** The International Physical Activity Questionnaire (IPAQ) was used to classify the participants into 3 groups: low, moderate, and high level of physical activity group, respectively. A total of 36 participants were recruited, 12 in each group. Participants were assessed for body composition, rest brachial-ankle pulse wave velocity (baPWV), handgrip strength (HGS), maximal oxygen uptake (V̇O_2max_), and MAET baPWV.

**Results:** In the three groups, significant differences were observed in V̇O_2max_, HGS, relative fat mass (%FM), body mass index (BMI), muscle mass ratio (MMR), and Rest baPWV (*p* < 0.05). After maximal aerobic exercise, the MAET baPWV values decreased significantly in all groups (all *p* < 0.001). Rest baPWV was significantly correlated with firefighters' age, seniority, metabolic equivalents (METs), height and muscle mass (MM) (*p* < 0.05).

**Conclusions:** Firefighters with high levels of physical activity had better body composition and physical fitness and lower Rest baPWV. In all three groups, baPWV was lower after the MAET than before it. Therefore, regardless of a firefighter's level of physical activity, high-intensity aerobic exercise may have a beneficial effect on arterial stiffness.

## Introduction

Firefighters encounter many dangerous conditions during their professional duties and must maintain physical standards to improve occupational safety and performance 1. Sufficient physical fitness improves the likelihood firefighters will successfully complete occupational tasks despite the constraints imposed by protective equipment (e.g., external firefighting gear, breathing apparatus) and the environment (e.g., increased temperature, smoke, carbon dioxide). Firefighting leads to significant cardiovascular strain, including alterations in cardiac function, vascular function, and hemostasis 2, leading to the danger of irreparable harm to the health of firefighters. A worrying situation is the high rate of overweight (BMI: 25.0-29.9 kg/m^2^) and obesity (BMI: ≥30.0 kg/m^2^) among firefighters, in which unsatisfactory physical fitness and obesity are associated with impaired work performance, increased risk of injury and duty-related sudden cardiac events 3-6.

Significant increases in the prevalence of overweight and obesity have been observed among firefighters in the United States, United Kingdom, and other developed countries, and many firefighters have also been noted to have one or more cardiovascular risk factors (such as obesity, hypertension, hyperlipidemia) and reduced aerobic capacity 7. However, according to a survey by the National Fire Protection Association (NFPA), less than 25% of US firefighters meet the amount of exercise recommended by the American College of Sports Medicine (ACSM) 8-10. According to the BMI data, approximately 50% of firefighters are overweight and 25% are obese 11, 12. Despite this, most fire agencies nationwide do not have routine, long-term physical training plans 11, 12. One of the main factors contributing to firefighters' obesity is their low levels of physical activity. Infrequent and non-occupational physical activity is limited (e.g., off the clock recreation, transportation, household) and may explain the observed BMI classifications 13.

Poor cardiorespiratory fitness (V̇O_2max_ below age-sex-matched inactive levels) 14, 15 and a low physical activity (less than 3 days of moderate/vigorous intensity activity per week) 16 have been identified as determinants of increased arterial stiffness 17. There is a similar association between sedentary physical activity and arteriosclerosis, and 13.4% of sedentary firefighters suffer from metabolic syndrome, which entails a higher risk of cardiovascular disease (CVD) factors in arteriosclerosis 18.

Sudden and intense physical exertion has been shown to trigger sudden cardiovascular events, especially in sedentary individuals 19, 20. Sudden cardiovascular events account for 45% of all firefighter deaths 21. In the past decade, prevention and response mechanisms have been actively used to reduce the occurrence of sudden cardiac death among firefighters, but it is still the number one killer of firefighters in the United States 21. The level of physical activity and cardiorespiratory fitness are inversely related to future CVD incidence; however, each additional metabolic equivalent of aerobic exercise may reduce the risk of mortality by 13% and CVD mortality by 15% 22.

The European Society of Cardiology guidelines for the management of pulse wave velocity (PWV) is the gold standard for the assessment of arteriosclerosis and can be used as an indicator of vascular damage, vascular compliance, and treatment effect 23, 24. Carotid-femoral PWV (cfPWV) 25 and brachial-ankle PWV (baPWV) 26 are the two most frequently applied PWV measurements. As a velocity, PWV can be conveniently measured as the distance divided by the time interval between two selected points of the arterial system 27. To measure cfPWV which is central arterial stiffness (carotid and femoral arteries), requires great skill and takes more than 20 minutes 28. A simpler method to determine baPWV which is peripheral arterial stiffness (brachial and ankle arteries) was developed in Japan, and the use of this procedure became widespread within a short time 28. Considering the unique work characteristics of firefighters and their health monitoring needs, the baPWV measurement method is more feasible. This technology is convenient, cost-effective, and suitable for rapid screening in various settings. Therefore, it is reasonable to support the current practice of using baPWV for monitoring the health of firefighters. A possible indicator of hardening blood vessels is baPWV greater than 1400 cm/s 26.

To our knowledge, there is currently a lack of relevant data on the impact of on-duty and off-duty physical activities (e.g., occupational, recreational, transportation, household, and exercise/leisure activities) and cardiopulmonary function on arterial stiffness in firefighters in Taiwan, and the degree of change in arterial stiffness (i.e. baPWV) after maximum intensity exercise also needs to be confirmed. Maximal intensity exercise directly impacts arteriosclerosis and blood vessel stiffness. By measuring baPWV before and after exercise, the immediate effects on vascular health can be assessed, which is particularly important for firefighters 29 or non-firefighters such as athletes 30. Therefore, this study aims to determine the relationships between the level of physical activity and arterial stiffness, between the level of physical activity and physical fitness performance, and between arterial stiffness and physical fitness performance in firefighters. We hypothesize that firefighters' levels of physical activity would associate with arterial stiffness and overall physical fitness, and that maximal intensity exercise will alter their arterial stiffness.

## Methods

### Study design

In this study, firefighters from different departments in northern Taiwan urban were grouped into three groups by level of physical activity, which according to the total physical activity volume reported on the International Physical Activity Questionnaire (IPAQ), using metabolic equivalents (METs) as the cut-off point 16. In order to reduce participants' self-report bias, the same experimenter assisted all participants in completing the IPAQ by explaining each question. The three groups were the low level of physical activity (LPAL, <600 MET-min/week), moderate level of physical activity (MPAL, 600-3,000 MET-min/week), and high level of physical activity groups (HPAL, >3,000 MET-min/week) 16. An arteriosclerosis tester (OMRON HBP-8000, Omron Healthcare Co., Ltd, Kyoto, Japan) was used to investigate differences in arterial stiffness among firefighters with different levels of physical activity and the effects following maximal intensity exercise.

### Participants

A total of 36 male firefighters (12 per group) who had passed the national examination and had been trained for more than one year voluntarily participated in this study. Participants were eligible if they were currently in active service and between 20 and 49 years of age. Participants were included if not on cardiovascular acting drugs. Exclusion criteria were those who had any cardiovascular disease or chronic disease diagnosed by a medical doctor, or smoker who smoked at least one cigarette per day. The participants could eat or drink 2 hours before the test, but they must avoid high-intensity exercise, caffeine and alcohol consumption in the 12 hours preceding the test. Sufficient sleep (≥ 7 hours) 31 is required the night before the test. The participants were not allowed to take any cardiovascular acting drugs. The sample size was estimated with a software program (G*Power v3.1.9.7; Heinrich-Heine-Universität Dusseldorf) with an effect size of 0.5, alpha error of 0.05, and statistical power of 0.8. Thus, the total sample size of participants required for the study was determined to be 33 (i.e., at least 11 participants per group). Before any experimental testing began, all participants completed informed consent.

### Procedures

**Figure 1:**
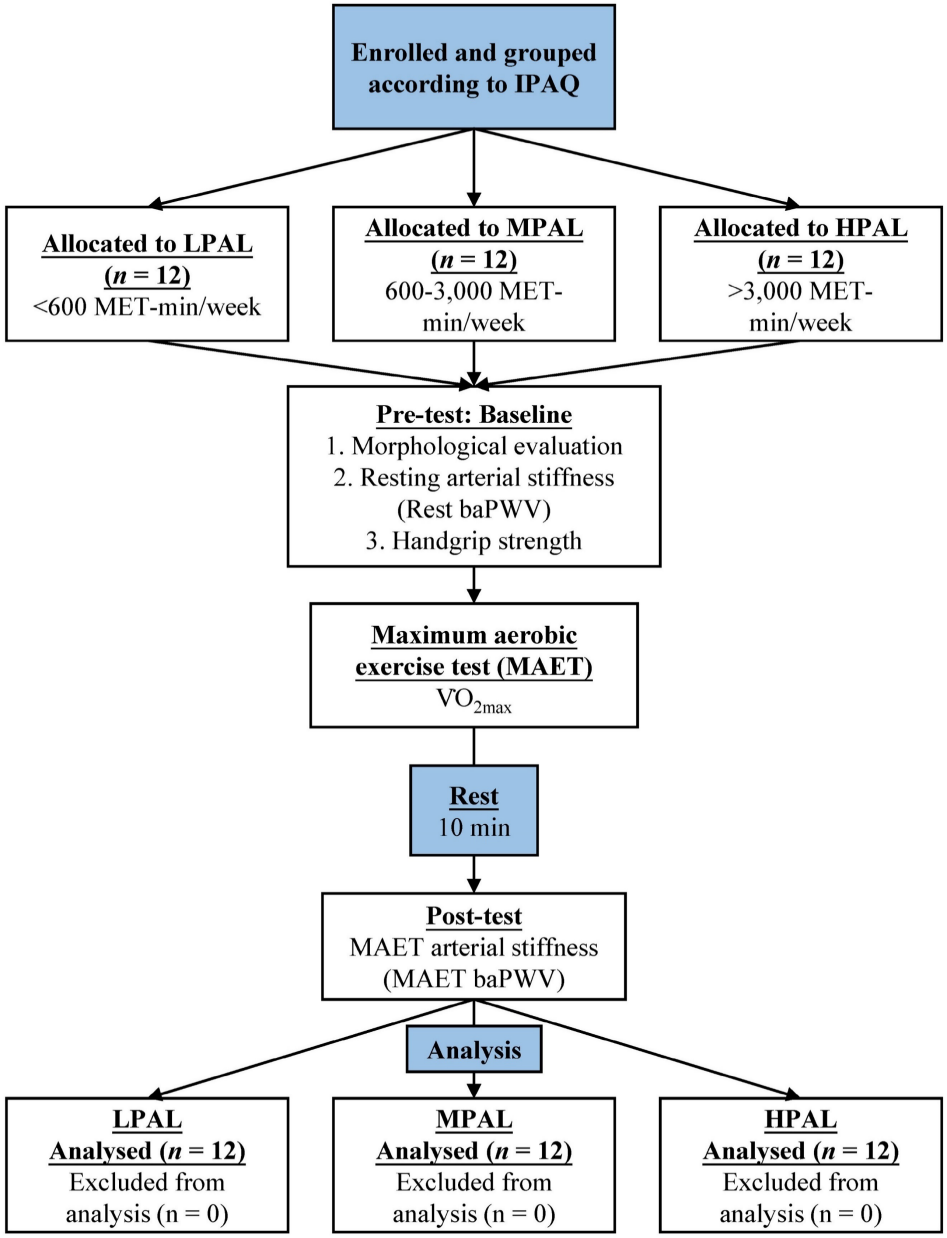
Experimental procedure description.

After the morphological evaluations, all participants were assessed for resting arterial stiffness, handgrip strength, maximum aerobic exercise test (MAET), and post-MAET arterial stiffness after 10 minutes of rest. The flow design of the study is shown in Fig. 1.

### Morphological evaluation

Morphological evaluations were assessed through standard procedures 32, 33. The weight, body mass index (BMI), relative fat mass (%FM), muscle mass (MM), and muscle mass ratio (MMR) of all participants were measured using a body composition analyzer (InBody 570, Biospace, Inc. Seoul, Korea), with additional inputs of their age and height measured by the height measurement equipment H900 (NAGATA Scale Co., Ltd. Tainan, Taiwan). The InBody^®^ 570 serves as a reliable method to estimate body composition with a multi-frequency bioelectrical impedance analyzer 32.

### Rest and post-MAET arterial stiffness testing

The data on baPWV were measured with an arteriosclerosis tester (OMRON HBP-8000, Omron Healthcare Co., Ltd, Kyoto, Japan) which with high reliability (ICC greater than 0.95) 34. The measurement of baPWV was performed by a trained examiner. The room temperature of the testing room was maintained at 22-25°C. Participants wore light short-sleeved sportswear and rested for > 5 minutes in a supine position on the massage table, and they were instructed to keep their bodies relaxed 35. Then the participants were asked to turn both palms upward by their sides. Four blood pressure cuffs were tied to the upper arms and ankles respectively, in accordance with established methods 35. The average values of the baPWV on the left and right sides was calculated 35. Two measurements were taken and if these values deviated by >5 mmHg, a third measurement was performed. The average of the two closest values was used 36.

Participants laid supine to passively rest for 10 minutes immediately following the MAET 37. Then, the examiner performed the same arterial stiffness test process again 35. For each measurement taken, the average baPWV on the left and right sides was calculated.

### Maximum handgrip strength (HGS) testing of dominant hand

A Takei 5401 handgrip digital dynamometer (Takei Scientific Instruments Co., Ltd, Tokyo, Japan) was used for HGS measurement. Participants stood in a standing position with their elbows straight and relaxed. When they heard the start command, they exerted maximum force for at least 3 seconds until the value on the handgrip strength indicator stopped increasing 37. The highest value of three measurements was taken as the final result.

### Maximal aerobic exercise test (MAET)

MAET was performed with the Cardiopulmonary Exercise Testing System (Vmax Encore 29 System, VIASYS Healthcare Inc, Yorba Linda, CA, USA). Before each test, the instrument was warmed up for at least 15 minutes before being calibrated. Participants wore a face mask (Hans-Rudolph) that covered their noses and mouths and a chest strap heart rate monitor (Polar H10, Polar Electro Oy, Finland). The initial speed of the treadmill was set at 7.2 km/h, and the speed increased by 1.8 km/h every 2 minutes until the participant was exhausted 39. Rate of Perceived Exertion (RPE) was evaluated every 2 minutes using a 6-20 category-ratio scale 40. The test was completed when the participants reached volitional exhaustion or met at least three of the following criteria 41, 42: 1) peak heart rate greater than 90% of predicted maximum heart rate (HR_max_ = 220 - age); 2) plateau in oxygen consumption (i.e., < 2.1 mL/kg/min increase in V̇O_2max_ with concurrent increase in workload); 3) respiratory exchange ratio > 1.1; and 4) RPE of 18-20. Maximal oxygen uptake (V̇O_2max_) was defined as the highest 20-second value achieved during a MAET.

### Statistical Analysis

All data are presented as mean ± standard deviation (SD). The statistical software IBM Statistical Package for the Social Sciences (SPSS) (version 20, IBM Corp., New York, NY, USA) was used for statistical analysis. The normal distribution of the data was analyzed by Shapiro-Wilks test. One-way analysis of variance (ANOVA) with post-hoc Bonferroni was used to test whether there was a significant difference in METs, morphological characteristics, and physical fitness between groups with different levels of physical activity. A 2 x 3 repeated-measures ANOVA examined the interaction between group (LPAL, MPAL and HPAL) and tests (Rest baPWV, MAET baPWV). To evaluate the differences between Rest baPWV and MAET baPWV, we compared the differences in baPWV of each group at rest and after MAET by a paired t-test. One-way ANOVA with post-hoc Bonferroni was used to assess the effects of baPWV in the three groups. Pearson's correlation coefficient was used to analyze the correlations between Rest baPWV and METs, age, seniority, morphological evaluations, and physical fitness parameters. The level of significance was set at *p* < 0.05.

## Results

### The basic characteristics of participants

All continuous data showed normal distributions (*p* > 0.05) by Shapiro-Wilks test, meeting the statistical assumptions of parametric tests. Table 1 presents total data and differences in physical activity (METs), age, seniority, morphological characteristics, and physical fitness in groups with different physical activity levels. The results showed significant differences in METs (F = 131.12, *p* < 0.001), weight (F = 3.70, *p* = 0.035), body mass index (BMI, F = 3.63, *p* = 0.037), relative fat mass (%FM, F = 17.90, *p* < 0.001), muscle mass ratio (MMR, F = 16.53,* p* < 0.001), and V̇O_2max_ (F = 20.28, *p* < 0.001) and handgrip strength (HGS, F = 3.71, *p* = 0.035). Specifically, according to the post-hoc results, METs was significantly higher in the HPAL than in the MPAL and LPAL; relative fat mass was significantly lower in the HPAL than in the MPAL and LPAG; BMI was significantly lower in the HPAL than in the LPAL; MMR was significantly higher in the HPAL than in the MPAL and LPAL; V̇O_2max_ was significantly higher in the HPAL than in the MPAL and LPAL, and also significantly higher in the MPAL than in the LPAL. Handgrip strength was significantly higher in the HPAL than in the LPAL. Rest baPWV was significantly lower in the HPAL than in the LPAL.

### Differences in baPWV and the acute effect of MAET in the three groups

The results of the 2 x 3 repeated-measures ANOVA for baPWV among the three groups are shown in Table 2. No statistically significant group 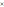 test interactions were found for LPAL, MPAL, and HPAL (F = 1.06, *p* = 0.364, ƞ^2^ = 0.09). A significant effect was observed in groups (F = 3.48, *p* = 0.049, ƞ^2^ = 0.24) and tests (F = 413.69, *p* < 0.001, ƞ^2^ = 0.97), respectively. Post-hoc comparisons using the Bonferroni method revealed a significant difference in Rest baPWV between the HPAL and LPAL (*p* = 0.018); with paired t-tests revealed that the MAET baPWV values ​​decreased significantly from Rest baPWV in LPAL (*p* < 0.001), MPAL (*p* < 0.001), and HPAL (*p* < 0.001), respectively.

### Correlation between the Rest baPWV, physical activity, age, seniority, physical fitness and morphological attributes

Table 3 shows the results of the correlation analysis between Rest baPWV, physical activity, age, seniority, physical fitness and morphological attributes. The results showed that Rest baPWV was positively correlated with age (r = 0.500, *p* = 0.002) and seniority (r = 0.538, *p* = 0.001), but negatively correlated with METs (r = -0.415, *p* = 0.012), height (r = -0.384, *p* = 0.021) and V̇O_2max_ (r = -0.334, *p* = 0.046). METs was positively correlated with V̇O_2max_ (r = 0.701, *p* < 0.001), handgrip strength (r = 0.385, *p* = 0.020) and MMR (r = 0.683, *p* < 0.001), but negatively correlated with weight (r = -0.382, *p* = 0.021), BMI (r = -0.372, *p* = 0.025) and relative fat mass (r = -0.697, *p* < 0.001). Seniority was positively correlated with BMI (r = 0.380, *p* = 0.022), but negatively correlated with V̇O_2max_ (r = -0.480, *p* = 0.006). V̇O_2max_ was positively correlated with MMR (r = 0.717, *p* < 0.001), but negatively correlated with weight (r = -0.641, *p* < 0.001), BMI (r = -0.620, *p* < 0.001) and relative fat mass (r = -0.730, *p* < 0.001).

## Discussion

The primary aim of this study was to evaluate whether different levels of physical activity affect morphological attributes, physical fitness, and Rest baPWV in firefighters. In addition, the impact of different levels of physical activity on MAET baPWV after MAET was also examined in firefighters. The current study found that only highly physically active firefighters met the NFPA recommended indicators for safe fire rescue work in terms of body composition and physical fitness (PBF ≤ 18%, V̇O_2max_ ≥ 42 ml/kg/min) 12, 29, 43, and they also had lower arterial stiffness. When the MAET was performed, arterial stiffness decreased in all three groups of firefighters.

Being overweight not only makes firefighters susceptible to CVD but may also lead to major cardiovascular events, such as myocardial infarction or fatal arrhythmia. Firefighters in Taiwan remain on call in the fire station for a long time. If they fail to develop exercise habits, they can easily develop low activity levels and a sedentary lifestyle. This study found that in terms of body composition, the difference between %FM and MMR were most significant between the different activity groups (*p* < 0.001). The HPAL had the lowest %FM (16.9 ± 4.1%) and the highest MMR (47.0 ± 2.4%), as well as better physical fitness performance. The NFPA maintains that, to perform fire rescue tasks safely and competently, firefighters need to pay equal attention to aerobic capacity and muscle strength training, their %FM should be optimized to less than 18% as much as possible, and they should have a V̇O_2max_ of at least 42 ml/kg/min 12, 29, 43. The results of this study showed that (i) the HPAL presented the NFPA's recommended values for %FM and V̇O2max; (ii) the MPAL presented the NFPA's recommended value for V̇O2max, but the same is not observed for %FM; and (iii) LPAL does not reach the values recommended by the NFPA for %FM and V̇O2max.

Research on the physical fitness of firefighters has confirmed that excessive body fat easily causes increases in body temperature and makes it difficult for heat to dissipate. This is not conducive to firefighters' disaster relief operations. It has also been reported that, for every additional year a firefighter's age increases, their body mass will also increase, for an average weight gain of 0.42 kg, BMI gain of 0.13 kg/m^2^, and %FM gain of 0.18% 44. Ras and Leach (2022) investigated the relationship between levels of physical activity and physical fitness factors in firefighters. They used questionnaires to collect data on levels of physical activity and found that age and BMI were important predictors of insufficient physical activity among firefighters 45. Corresponding to the results of this study, their results showed that working experience had a strong positive correlation with BMI, while differences in physical activity had a strong negative correlation with %FM and a strong positive correlation with MMR. However, age was not found to have correlations with %FM or MMR, suggesting that, as age increases but the level of physical activity is maintained, physical fitness can still be maintained above the levels recommended by the NFPA. The most urgent task in fire service is fire rescue. Research by Nazari et al. (2018) pointed out that, for the fire hose task in fire service, the correlation between firefighters' V̇O_2max_ and HGS was the highest. In addition, they also found a correlation between the performance of weight-bearing ladder climbing tasks and V̇O_2max_ 46. It can be seen that high V̇O_2max_ and MMR levels can improve task efficiency and performance, and physical fitness levels can be maintained through physical training as age increases.

In Taiwan, in addition to fighting fires, members of the fire department also have additional responsibilities, such as emergency medical rescue, fire safety inspections, and document processing. This workload also leads to a lack of time for exercise, hypertension, dyslipidemia, and obesity, thereby increasing the incidence of CVD. Ras et al. (2022) pointed out that during their careers, firefighters will encounter many risk factors, which will have negative effects on the body and cause accidental injuries. The highest proportion of deaths is caused by CVD stemming from a lack of exercise and overwork, which increases cardiovascular damage, accounting for 45% of the causes of death 45. Gendron et al. (2020) conducted a study on firefighters' levels of physical activity and cardiovascular health indicators in Quebec, Canada. The results showed that firefighters who engaged in physical training while on duty had better cardiovascular health markers than those who did not 47.

Arterial stiffness is considered an important risk factor for CVD and can predict adverse cardiovascular outcomes 48, 49. Therefore, assessing arterial stiffness may facilitate the identification of early-stage vascular diseases 50. The average Rest baPWV value of participants in this study was 1271.6 ± 68.8 cm/s, but the analysis also showed that Rest baPWV was not significantly related to V̇O_2max_. In addition, Rest baPWV had a strong positive correlation with age and work seniority. There were significant differences in Rest baPWV between the three groups, with Rest baPWV significantly higher in the LPAL than in the HPAL. A baPWV result greater than 1400 cm/s indicates that blood vessels may tend to harden 25. The average Rest baPWV value of LPAL was 1310.4 ± 80.4 cm/s, which is close to the recommended warning threshold of 1400 cm/s. It is necessary to pay attention to the subsequent tendency of blood vessels to harden.

This study showed that arterial stiffness decreased in all three groups after 10 minutes of MAET. Arterial stiffness significantly decreased by 14% in the HPAL, by 14% in the MPAL, and by 15% in the LPAL. In related literature, research by Santos et al. (2023) found that young and lean firefighters with high aerobic fitness showed significant decreases in arterial stiffness after simulated fire rescue operations and maximum aerobic exercise 51. Niebauer et al. (2020) pointed out that a low-activity or sedentary lifestyle is likely to increase PWV and the risk of CVD. In that study, PWV was measured before exercise and 10 minutes after maximum intensity exercise. It was found that higher exercise intensity will lead to a decrease in PWV, while exercise intensity not exceeding 50% V̇O_2max_ will not reduce PWV. Furthermore, in terms of the hemodynamic mechanism, high-intensity aerobic exercise will release higher amounts of nitric oxide, leading to greater relaxation of smooth muscle, followed by a further reduction in arterial stiffness 37. In conclusion, the current study showed that the primary factors affecting baPWV are age, seniority, and level of physical activity. The group with the highest level of physical activity had the lowest Rest baPWV values, indicating better blood vessel elasticity. Ten minutes after high-intensity exercise in each group, decreases in baPWV were noted. However, the duration of such decreases in baPWV values ​​will require follow-up research to confirm.

## Limitations

This study has several limitations. First, this study may be limited by potential selection bias, namely the lack of female participants, who were not included in the current study because males predominantly constitute the local firefighter population. Therefore, these results may not be applicable to the physical fitness and cardiovascular health of female firefighters. Another limitation is that the firefighters tested in this study used a treadmill to measure their maximum aerobic exercise capacity in a fixed-temperature laboratory, which may not truly simulate the fatigue and physiological parameters produced during firefighting and disaster relief. Therefore, future studies should include larger sample sizes to confirm potential changes in arterial stiffness due to different health conditions, morphological and fitness attributes in both male and female firefighters.

## Conclusions

The current study found that only highly active firefighters met the NFPA-recommended indicators for safe fire rescue work in terms of morphological attributes and physical fitness, and they also had lower arterial stiffness. When the maximum aerobic exercise test was performed, arterial stiffness decreased in all three groups of firefighters. The current study suggests that firefighters with low and medium activity levels should improve their activity levels and physical fitness to ensure safety and efficiency at work. Those of older age and longer working seniority should pay attention to the possibility of reductions in blood vessel elasticity. It is recommended that appropriate and periodized high-intensity physical training be implemented to reduce arterial stiffness and the incidence of arteriosclerosis, strengthening the total capability of disaster relief personnel while ensuring the health and safety of firefighters and protecting people's lives and property.

## Figures and Tables

**Figure 2:**
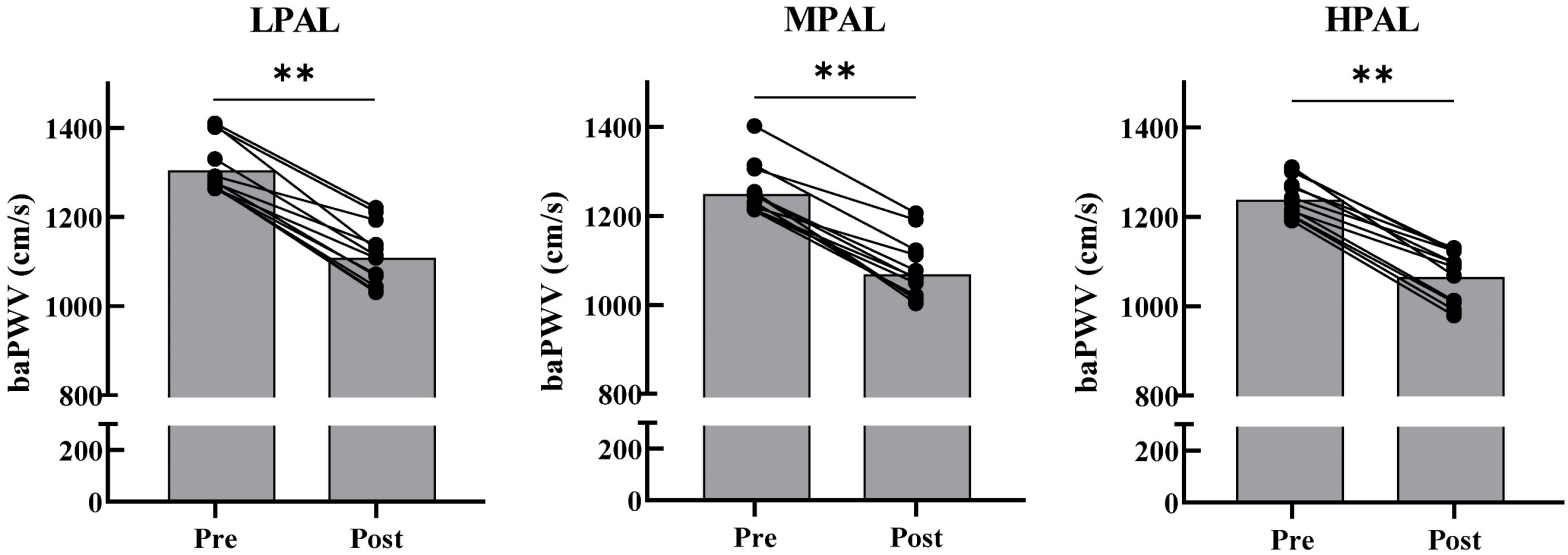
**Changes in baPWV after maximal aerobic exercise testing for LPAL, MPAL, and HPAL.** LPAL, low level of physical activity group. MPAL, moderate level of physical activity group. HPAL, high level of physical activity group. Gray bars represent the group mean. Solid black dots represent individual data points at pre and post-test, respectively. ** Indicates post-test was significantly difference from pre-test, *p* < 0.001.

**Table 1:** METs, morphological characteristics, and physical fitness of participants in the three groups.

	All groups	LPAL	MPAL	HPAL	*p*-Value
	n =36	n = 12	n = 12	n = 12	
**METs**	1909.8 ± 1383.7	434.7 ± 132.1†‡	1726.3 ± 648.0†	3568.3 ± 493.5	<0.001
95% CI		350.8-518.6	1314.6-2138.1	3254.7-3881.8	
**Age (years)**	34.7 ± 5.9	36.0 ± 6.9	32.3 ± 5.8	35.8 ± 4.8	0.238
95% CI		31.6-4.04	28.7-36.0	32.8-38.8	
**Seniority (years)**	12.0 ± 6.5	14.1 ± 7.1	10.8 ± 6.1	11.1 ± 6.1	0.401
95% CI		9.56-18.6	6.9-14.7	7.2-15.0	
**Height (cm)**	176.2 ± 4.4	177.1 ± 4.4	176.3 ± 4.3	175.1 ± 4.7	0.546
95% CI		174.3-179.9	173.6-179.1	172.1-178.1	
**Weight (kg)**	80.2 ± 10.4	84.9 ± 11.6†	81.3 ± 8.5	74.4 ± 8.5	0.035
95% CI		77.6-92.3	75.9-86.8	69.0-79.8	
**BMI (kg/m^2^)**	25.8 ± 2.8	27.1 ± 3.3†	26.1 ± 1.8	24.3 ± 2.5	0.037
95% CI		25.0-29.2	24.9-27.2	22.7-25.8	
**%FM (%)**	23.3 ± 6.7	28.3 ± 6.2†	24.7 ± 3.6†	16.9 ± 4.1	<0.001
95% CI		24.4-32.3	22.4-27.0	14.3-19.5	
**MM (kg)**	35.5 ± 3.2	34.2 ± 3.9	34.6 ± 2.7	34.9 ± 3.0	0.881
95% CI		31.7-36.7	32.9-36.2	33.0-36.8	
**MMR (%)**	43.4 ± 3.9	40.5 ± 3.6†	42.6 ± 2.3†	47.0 ± 2.4	<0.001
95% CI		38.2-42.8	41.2-44.1	45.5-48.5	
**V̇O_2max_ (ml/kg/min)**	43.9 ± 7.0	37.6 ± 5.8†‡	44.0 ± 3.8†	50.1 ± 4.6	<0.001
95% CI		33.9-41.3	41.6-46.4	47.1-53.0	
**HGS (kg)**	47.9 ± 4.6	45.2 ± 3.9†	49.1 ± 4.8	49.5 ± 4.1	0.035
95% CI		42.7-47.7	46.1-52.2	46.9-52.1	

All data are presented as means ± SD. LPAL, low level of physical activity group. MPAL, moderate level of physical activity group. HPAL, high level of physical activity group. METs, metabolic equivalents. Seniority, work seniority. BMI, body mass index. %FM, relative fat mass. MM, muscle mass. MMR, muscle mass ratio. V̇O_2max_, maximal oxygen uptake. HGS, handgrip strength. † Significantly difference from HPAL, *p* < 0.05. ‡ Significantly difference from MPAL, *p* < 0.05.

**Table 2:** Measures results obtained Rest baPWV and MAET baPWV with different levels of physical activity groups.

	LPAL	MPAL	HPAL	*p* Group	*p* Test	*p* Group 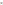 Test
	n = 12	n = 12	n = 12			
**Rest baPWV (cm/s)**	1310.6 ± 60.5†	1258.8 ± 56.5	1245.8 ± 43.5	0.049	<0.001	0.364
95% CI	1272.1-1349.1	1222.9-1294.6	1218.1-1273.4			
**MAET baPWV (cm/s)**	1113.7 ± 67.6**	1077.5 ± 68.1**	1070.5 ± 56.9**			
95% CI	1070.7-1156.6	1034.2-1120.8	1034.4-1106.6			

All data are presented as means ± SD. LPAL, low level of physical activity group. MPAL, moderate level of physical activity group. HPAL, high level of physical activity group. baPWV, brachial-ankle pulse wave velocity. MAET, maximal aerobic exercise test. † Significantly difference from HPAL, *p* < 0.05. ** Indicates MAET baPWV was significantly difference from Rest baPWV, *p* < 0.001.

**Table 3:** Correlations between Rest baPWV, physical activity, age, seniority, physical fitness and morphological attributes.

	n = 36										
	baPWV	METs	Age	Seniority	VO_2max_	HGS	Height	Weight	BMI	MM	MMR
METs	-0.415*	-									
Age	0.500**	0.020	-								
Seniority	0.538**	-0.142	0.904**	-							
V̇O_2max_	-0.334*	0.701**	-0.323	-0.448**	-						
HGS	-0.159	0.385*	-0.178	-0.116	0.171	-					
Height	-0.365*	-0.193	-0.053	-0.134	-0.299	-0.156	-				
Weight	-0.067	-0.382*	0.173	0.275	-0.641**	0.050	0.554**	-			
BMI	0.103	-0.372*	0.227	0.380*	-0.620**	0.126	0.207	0.928**	-		
MM	-0.324	0.125	0.135	0.149	-0.189	0.317	0.552**	0.698**	0.565**	-	
MMR	-0.233	0.683**	-0.104	-0.245	0.717**	0.247	-0.241	-0.702**	-0.729**	0.012	-
%FM	0.234	-0.697**	0.101	0.251	-0.730**	-0.226	0.241	0.732**	0.763**	0.034	-0.997**

baPWV, brachial-ankle pulse wave velocity. METs, metabolic equivalents. V̇O_2max_, maximal oxygen uptake. HGS, handgrip strength. BMI, body mass index. MM, muscle mass. MMR, muscle mass ratio. %FM, relative fat mass. * Correlation is significant at the 0.05 level (2-tailed). ** Correlation is significant at the 0.01 level (2-tailed).

## Abbreviations

ACSM: American College of Sports Medicine
AS: arterial stiffness
ANOVA: analysis of variance
baPWV: brachial-ankle pulse wave velocity
BMI: body mass index
CV: cardiovascular
CVD: cardiovascular disease
HGS: handgrip strength
HR: heart rate
HPAL: high level of physical activity group
IPAQ: International Physical Activity Questionnaire
LPAL: low level of physical activity group
MAET: maximal aerobic exercise test
METs: metabolic equivalents
MetS: metabolic syndrome
MPAL: moderate level of physical activity group
NFPA: National Fire Protection Association
PA: physical activity
PAL: physical activity level
%FM: relative fat mass
PF: physical fitness
PWV: pulse wave velocity
RPE: Rate of Perceived Exertion
MM: muscle mass
MMR: muscle mass ratio
SD: standard deviation
V̇O_2max_: maximal oxygen uptake
